# Perioperative Considerations for the Surgical Treatment of Crohn’s Disease with Discussion on Surgical Antibiotics Practices and Impact on the Gut Microbiome

**DOI:** 10.3390/antibiotics13040317

**Published:** 2024-03-30

**Authors:** Shelbi Olson, Lindsay Welton, Cyrus Jahansouz

**Affiliations:** 1 Department of Surgery, University of Minnesota, Minneapolis, MN 55455, USA; olso9795@umn.edu (S.O.); welto031@umn.edu (L.W.); 2 Division of Colon and Rectal Surgery, University of Minnesota, Minneapolis, MN 55455, USA

**Keywords:** Crohn’s disease, *Mycobacterium paratuberculosis*, IBD, microbiome, bacteria, antibiotics, mycobacteria, surgery

## Abstract

Crohn’s disease, a chronic inflammatory process of the gastrointestinal tract defined by flares and periods of remission, is increasing in incidence. Despite advances in multimodal medical therapy, disease progression often necessitates multiple operations with high morbidity. The inability to treat Crohn’s disease successfully is likely in part because the etiopathogenesis is not completely understood; however, recent research suggests the gut microbiome plays a critical role. How traditional perioperative management, including bowel preparation and preoperative antibiotics, further changes the microbiome and affects outcomes is not well described, especially in Crohn’s patients, who are unique given their immunosuppression and baseline dysbiosis. This paper aims to outline current knowledge regarding perioperative management of Crohn’s disease, the evolving role of gut dysbiosis, and how the microbiome can guide perioperative considerations with special attention to perioperative antibiotics as well as treatment of *Mycobacterium avium* subspecies *paratuberculosis*. In conclusion, dysbiosis is common in Crohn’s patients and may be exacerbated by malnutrition, steroids, narcotic use, diarrhea, and perioperative antibiotics. Dysbiosis is also a major risk factor for anastomotic leak, and special consideration should be given to limiting factors that further perturb the gut microbiota in the perioperative period.

## 1. Introduction: Incidence, Etiology, Overview of Surgical Management, and Aims of This Paper

Crohn’s disease (CD) is a chronic inflammatory bowel disease (IBD) characterized by transmural inflammation affecting any portion of the gastrointestinal tract, most commonly involving the terminal ileum. Classically, symptoms and flares relapse and remit, and sequelae include fistula, abscesses, anal fissures, ulcers, obstruction, stricturing, malnutrition, and neoplasia. IBD disproportionately affects North Americans and Europeans with a prominent north–south gradient. However, over the last several decades there has been a marked increase in the incidence of IBD across a more geographically diverse population and is now considered a global disease [1,2,3]. Coward et al. postulate the prevalence of IBD will increase by 2.86% per year in Canada, and by 2030 3.48 million people in the US alone will be living with IBD [4]. As the prevalence increases, it poses a steadily evolving burden on worldwide healthcare systems. IBD is associated with poor quality of life, morbidity, extended hospital stays, multiple operations, and significant overall cost of disease [5].

Despite the significance of CD, the true etiopathogenesis is unclear. It is likely multifactorial and combines a genetic predisposition, environmental exposures, microbial and metabolic factors, among others. Central to most accepted theories is inflammation and injury to the gastrointestinal tract, which continues unchecked, leading to mucosal destruction and, with time, chronic inflammatory sequelae. However, the initial insult or trigger of this unregulated inflammation is unclear and likely varies between individuals.

Roughly 200 genetic foci have been linked to IBD [6]. The greatest known risk factor is having a close relative with IBD [1,7], and there is an increase in disease incidence on an individual and societal level as communities progress economically [1,2,3,8]. Some research suggests that the use of antibiotics early in life, antibiotics in food, and an overall decrease in exposure to pathogens, as described in the hygiene hypothesis, contribute to an increased risk of dysregulated immune responses later in life. Other theories point to the Western diet, which is proinflammatory, leading to changes in the gut microbiome composition [2,9]. Another long-standing hypothesis is that various microorganisms can trigger IBD. In particular, it has been hypothesized for decades that Mycobacterium avium subspecies paratuberculosis (MAP) is a trigger of CD [1]. It is suggested that the common denominator tying many of the leading theories together is the role of the gut microbiome and how disturbances in gut microbiome homeostasis, termed “gut dysbiosis”, play a key role. The relative abundance of multiple bacteria has been implicated, including *Firmicutes* and *Proteobacteria*, but a consistent pattern of gut microbial change is not seen across CD patients. How gut dysbiosis contributes to the disease manifestation, or whether CD results in dysbiosis or both, is debated [1,10]. Mounting evidence suggests that dysbiosis is a causative agent in CD via reduction of anti-inflammatory mechanisms and induction of proinflammatory processes in the gut [11]. Short-chain fatty acid (SCFA) production is of particular interest, as it is a major energy source for enterocytes and provides upregulation of anti-inflammatory activities [12]. 

Understanding the optimal treatment and possible cure for CD centers on understanding the true etiopathology, and until then, surgery will likely play a central role in treatment and symptom management. The role of surgery for CD has evolved over time, especially with breakthroughs in medical management. Additionally, the role of surgery is highly dependent on the location of the disease along the gastrointestinal tract, the severity of diseases, the length of affected tissue, the presence of perianal disease, and if the disease is stricturing, penetrating, both, or neither. Although in exceptional circumstances, such as when the disease is solely ileocecal in nature and surgical resection can be functionally “curative”, it is important to note that most of the time, surgery in CD patients should be avoided. Surgery is not curative and is used largely when medical management has been exhausted. With that in mind, three out of four patients with CD will ultimately undergo surgical resection, and fifty percent of patients who require an initial surgical intervention will require a subsequent operation [3,12,13]. The predicted incidence of surgical intervention in this population highlights the importance of a multidisciplinary approach to patients with CD, as it enables careful disease monitoring and forward-thinking surgical management. Indications for surgery include severe stricture, abscess, perforation, sepsis, uncontrolled symptoms with treatment-resistant disease, and malignancy. Following patients closely allows surgery to be planned in advance and efforts to minimize the risk of complications such as perforation. Elective surgery, once a patient is optimized, allows the greatest potential for preservation of the bowel, the cornerstone of surgery in CD. Surgical options are driven by the dominating disease pathology and presentation, but for intra-abdominal disease, include resection: removing the diseased portion of bowel; stricturoplasty: widening the bowel in an effort to remove pathologic and symptomatic narrowing while preserving length; and fistulectomy: removing pathologic connections between the bowel and surrounding organs or the skin. A consideration of particular interest is the effect that surgery has on the microbiome and how this affects disease progression and surgical outcomes. 

There are currently many unanswered questions, including the interplay of CD and gut dysbiosis as well as the effects of surgery and perioperative management on the microbiome. CD patients are more susceptible to disturbances that may lead to unnecessary complications, and the perioperative period is fraught with interventions and treatments that can exacerbate dysbiosis. The complex nature of CD pathophysiology and patients’ immunocompromised status make complications in this patient population particularly devastating. It is critical that current knowledge surrounding these questions is synthesized to provide organization and direction for areas of future investigation and tailored perioperative care. Therefore, in this article, we outline current considerations for perioperative CD management with a focus on the evolving role of gut dysbiosis and how the microbiome can help guide perioperative management, how antibiotics, especially in the preoperative and bowel preparation context, may influence disease pathogenesis and highlight particular considerations for MAP.

## 2. Materials and Methods

A search of the literature was completed using the keywords Crohn’s Disease, microbiome, *Mycobacterium avium paratuberculosis*, surgery, bowel preparation, and antibiotics. Relevant articles were collected and reviewed by all authors to determine relevance for inclusion. Images included were designed by the authors and created using BioRender.com (accessed on 20 February 2024).

## 3. Overview of the Microbiome in Crohn’s Disease

The gut microbiota is incredibly diverse and varies naturally within an individual person both over time as well as between anatomic locations in their gastrointestinal tract. The gut microbiome has been mapped in efforts to establish patterns that can be defined as typical for both healthy individuals as well as specific disease states. Broadly, over 2100 species spanning 12 phyla are characteristic of the human microbiome. Despite this range, over 90% of human gut bacteria are classified into the following four phyla: *Proteobacteria*, *Actinobacteria*, *Firmicutes*, and *Bacteroidetes*. The number of commensal enteric bacteria is at its greatest concentration in the ascending colon at roughly 10^11,12^ cells/gram compared to only 10^2,3^ in the jejunum and proximal ileum [14,15,16]. Gut dysbiosis is now believed to be linked to innumerable disease processes ranging from gastrointestinal diseases to neurologic disorders [17,18]. 

**Figure 1:**
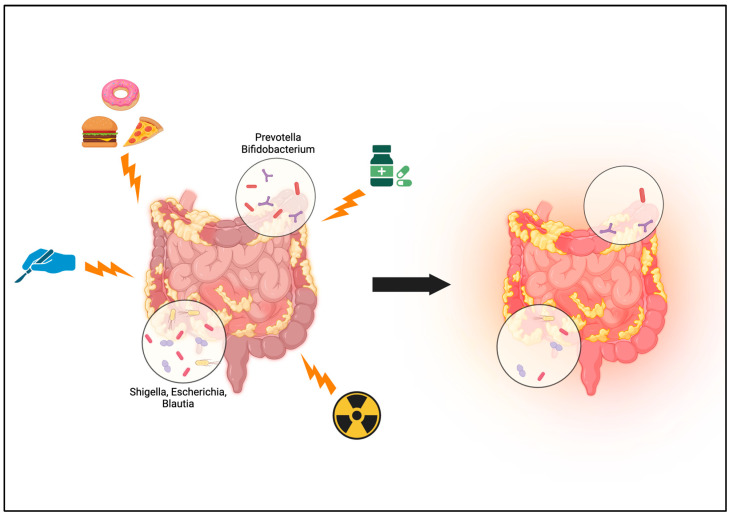
Numerous factors influence gut dysbiosis in CD patients, including medications, antibiotics, Western diet, radiation, and surgery. Repeated disruptions to the volatile microbiome of a CD patient can lead to further mucosal damage, inflammation, and dysbiosis. The microbiome across the colon in CD patients varies, and it is suggested that *Escherichia*, *Shigella*, and *Blautia* are associated with terminal ileal disease, while *Prevotella* and *Bifidobacterium* are linked to primary colonic CD. Original graphic designed by authors. Created with BioRender.com (accessed on 20 February 2024).

It is hypothesized that gut dysbiosis, specifically a decrease in bacterial diversity, is associated with CD. Notably, *Firmicutes* and *Bacteroides* are decreased, and *Enterobacteriaceae* is proportionally higher in CD patients [17]. A critical role played by bacteria in the human gut is the production of SCFA through fermentation. SCFA are used for energy by enterocytes and are critical to gut and immune homeostasis. *Faecalibacterium*, *Roseburia*, *Eubacterium*, *Clostridium*, and *Fusobacterium* all produce the SCFA butyrate; therefore, in states of dysbiosis where these bacteria are depleted, SCFA production is affected. Understandably, depletion in SCFA can then be linked to dysregulated immune function, intestinal inflammation, and poor healing [12]. Research has also suggested that the intestinal location of CD is linked to specific patterns of microbiome disturbances. Boatman et al. found *Escherichia*, *Shigella*, and *Blautia* are associated with terminal ileal disease in contrast to primary colonic CD associated with *Prevotella* and *Bifidobacterium* [17] [Figure 1]. This study’s main limitation was the sample size of only nine patients with active CD age-matched with seven controls; however, their results support the hypothesis that disease-specific microbiome patterns, or signatures, exist and can be measured. Understanding the baseline gut dysbiosis in CD patients is critical, as innumerable factors, including perioperative antibiotics, bowel preparation, diet, radiation, medication, and surgery itself, can impact gut dysbiosis. This can be particularly devastating in CD patients as distortions of gut microbiota are hypothesized to be linked to further mucosal damage, poor healing, and anastomotic leaks [Figure 1]. Blakeley-Ruiz et al. found that patients with IBD exhibited more volatility in microbiome composition over time as compared to healthy individuals with an increase in dysbiosis postoperatively [19]. Furthermore, dysbiosis has been linked to an increased risk of anastomotic leak [17], which can be a catastrophic complication of colorectal surgery. Disturbances in the gut microbiome and association with anastomotic leaks is a concept that dates back to the 1950s. Recent research suggests that low levels of *Faecalibacterium* are correlated to anastomotic leak rates. Further studies have shown differences in ileal microbiota and microbial-associated inflammatory factors when comparing initial surgery in CD patients to relapse and second surgery [20]. 

## 4. *Mycobacterium avium paratuberculosis*

Since the idea was introduced in 1913, MAP as a causative agent of enteritis, and specifically CD, has been debated with no clear consensus [21,22]. Arguments against the causative nature center on patterns of disease and treatments. Multiple experts point out that if MAP was causative of CD, TNF therapies should worsen the disease and not improve it. Epidemiologically, there should also be a higher incidence of CD in individuals exposed to animals susceptible to MAP [22,23,24]. There is compelling evidence of an association between CD and MAP but a causal association is difficult to show for many reasons: the bacteria is difficult to culture, polymerase chain reaction (PCR) is difficult due to a thick cell wall, and there are challenges with buoyancy as related to centrifugation [23,24]. However, a meta-analysis by Feller et al. found a strong association but significant heterogeneity between studies [25]. Because of the connection, anti-MAP therapies are being tested as a treatment for CD. A two-year study of clarithromycin, rifabutin, and clofazimine showed effectiveness in disease activity at 16 weeks, but not long-term remission [26]; however, a later analysis of the same dataset using intent-to-treat methodology did find durable benefit [27]. Patients who are eligible for anti-MAP are usually severe cases unresponsive to currently available therapeutics and, therefore, represent only a subset of the population of interest [22]. However, as will be discussed later, there may be microbiota data that is predictive of response to specific treatments. Perhaps there is a role for anti-MAP therapy in patients who have predictive markers of treatment failure even before they undergo other treatments.

In addition to direct treatment of MAP with antimicrobial agents, 5ASA also inhibits the growth of MAP in culture [28]. The benefit of mesalamine and related medications on CD may be twofold: decreasing inflammation and inhibiting MAP proliferation. However, the true pathogenicity of MAP in the Crohn’s gut, and therefore the impact of medications that reduce MAP activity, remains unknown.

**Figure 2:**
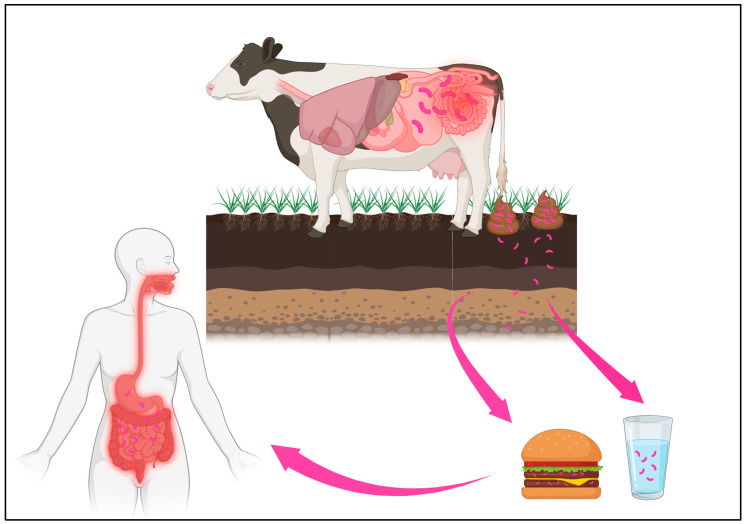
MAP is hypothesized to cause CD in part because it causes a form of enteritis in cows, termed Johne’s disease. Affected cows pass MAP into their stool and the bacteria can subsequently be detected in the surrounding soil, water, as well as milk and meat from the cows. This can lead to repeated exposures in humans. It is postulated that in susceptible individuals, MAP contributes to immune dysregulation, gut inflammation, and CD. Original graphic designed by authors. Created with BioRender.com (accessed on 20 February 2020).

The role of MAP in CD overall is poorly understood [Figure 2]. However, patients presenting for surgical intervention may be currently on or have previously received anti-MAP therapy. Particularly because surgical indications include disease refractory to medical therapy, this population is more likely to be eligible for MAP antibiosis. Broad-spectrum antibiotics are known to have dysbiotic consequences, sometimes long-term [29]. Regardless of treatment target, anti-MAP therapy, or other antibiotics, the gut of patients with CD is likely to see multiple exposures to antibiotics throughout a lifetime, and therefore, they may have many exacerbations of their dysbiosis. Because of the surgical risks related to dysbiosis, careful consideration of antibiotics for MAP treatment or for complications such as fistula and abscess should be exercised throughout a CD patient’s lifetime. It is prudent to minimize factors that will worsen or extend dysbiosis when possible since this population is likely to have an operation at some point.

## 5. Perioperative Optimization

Hospitals and surgeons have attempted to improve outcomes and decrease length of stay postoperatively in a variety of ways. Two of the most widely accepted approaches include prehabilitation and enhanced recovery after surgery (ERAS) protocols [30,31]. Prehabilitation is a multidimensional approach that aims to engage patients in the preoperative period to prepare for surgery through lifestyle modifications and education in hopes of optimizing them before surgery as opposed to simply risk-stratifying them in the preoperative period. Though this concept is exciting, evidence has been variable in proving effectiveness on typical measured postoperative outcomes, likely due to study design and population heterogeneity. However, components of prehabilitation have shown promise in patients with comorbidities, decreased functional status, frailty, malnutrition, and cancer [32,33,34,35,36,37]. In 2017, the American Society of Colon and Rectal Surgeons (ASCRS), along with the Society of American Gastrointestinal and Endoscopic Surgeons (SAGES), published a clinical practice guideline regarding aspects of enhanced recovery protocol (ERP) for patients undergoing elective colorectal surgery. Strong recommendations from ERAS include a preoperative clear liquid diet until two hours before anesthesia induction and immediate resumption of regular diet postoperatively [30]. Neither prehabilitation nor ERAS approaches are standardized, and protocols vary by institution, but some of the most evidence-based components of both include nutrition, medication management, smoking cessation, pain management, and psychological health with a focus on anxiety and depression [30].

### 5.1. Nutrition

Appropriate nutrition can have profound impacts on the postoperative course, especially in patients who are malnourished, like many with CD. It decreases infection, as well as hospital and ICU length of stay [38]. Addressing nutrition prior to an operation enhances the ability of the body to respond to surgical insults. Traditionally, patients were told to remain NPO starting at midnight the night prior to surgery to reduce the risk of aspiration related to anesthesia and intubation [30]. Bowel rest in other scenarios increases the risk of ileus, mucosal atrophy, and bacterial translocation [39] and likely applies in the preoperative scenario as well. Therefore, maintaining PO intake as long as possible is crucial. Newer recommendations state that clear liquids, including those with carbohydrate supplementation, are allowed until two hours prior to surgery; with this, patients have better outcomes, maintain body mass and strength, and have decreased protein losses postoperatively [30,31].

In the postoperative phase, an oral diet is recommended immediately [30]. Patients with inflammatory bowel disease frequently present for surgery in a malnourished state for various reasons, including malabsorption, fast transit time, gastrointestinal losses, or fistulae bypassing absorptive intestine [40]. It is usually impossible to mitigate these issues before surgery as they are often the indication for operative intervention. Therefore, it is imperative for CD patients to minimize the time of inadequate nutrition in the perioperative period. Feeding hastens recovery of the gastrointestinal system and speeds up hospital discharge [30,41]. While this has not been studied specifically in Crohn’s patients, a few studies did include IBD patients.

### 5.2. Hydration

In addition to malnutrition, CD patients struggle with dehydration. According to ERAS protocol, clear fluids should be allowed up to two hours prior to anesthesia [31]. This was a change in practice from avoiding all food and liquid starting at midnight prior to surgery. Fluid intake until closer to the operation is safe, increases patient comfort [31], and may improve hydration status intra- and postoperatively. Again, this is especially important in patients with greater baseline preoperative risk of malnutrition, dehydration, and electrolyte imbalances.

### 5.3. Smoking Cessation

Tobacco significantly increases perioperative risk: smokers have a higher risk of infection, poor wound healing, and decreased cardiopulmonary function compared to non-smokers. Longer time to recovery and respiratory complications are also associated with exposure to secondhand smoke [42]. In addition, smoking is a known risk factor for worsening intestinal inflammation in CD and may impact MAP in the gut. In one experiment, a metabolite of nicotine increased the growth of MAP [43]. The authors propose this as a mechanism for increased disease severity related to smoking [43]. If MAP is involved in CD, the increase of MAP in smokers further underscores the importance of the avoidance of cigarettes in this patient population. In the preoperative setting, smoking should be stopped four weeks in advance if possible [44]. Given that nicotine may have a direct role in disease severity, specifically in Crohn’s patients, short-term nicotine replacement therapy in the perioperative setting may not be a great alternative, and patients should be advised to quit smoking altogether.

### 5.4. Psychological Health and Stress

Anxiety increases negative outcomes and length of stay in addition to worsening immune function, wound healing, and functional return to baseline. Patient engagement and motivation are also adversely affected [45]. There is increasing evidence that implicates the relationship between the brain and the microbiome of the gut [46]. For example, cytokine release in response to psychological stressors has been linked to gut microbiota [47], and the introduction of pathogenic bacteria results in vagal activation even without producing inflammation in the intestine [48]. Additionally, Sudo et al. demonstrated a reduction in HPA axis levels back to normal after reconstitution of the microbiota [49]. In a chronically ill patient population at risk for psychological stress, it is imperative to include mental health providers in the team-based approach to caring for these patients in order to minimize the risk of postoperative complications and dysbiosis.

## 6. Preoperative Bowel Preparation

Standard of care in the United States includes surgical bowel preparation, defined as the combination of mechanical and oral antibiotics in preparation for colorectal surgery [50]; however, there is mixed evidence for their use. The ASCRS and SAGES give the use of preoperative bowel preparation a weak recommendation [30], and the joint guidelines from the ERAS Society, International Association for Surgical Metabolism and Nutrition (IASMEN), and the European Society for Clinical Nutrition and Metabolism (ESPEN) give a strong recommendation against routine mechanical bowel preparation [31]. Continued use of antibiotic and mechanical bowel preparation is likely related to the tracking of quality metrics such as postoperative infection and surgical site infection (SSI), causing surgeons and hospitals to be particularly focused on reducing these risks. The rate of infection in colon surgery with no antibiotic prophylaxis is likely around 30–40% [51], and a 2014 Cochrane Review by Nelson et al. found a clear benefit of antibiotics compared to none or to placebo in regards SSI risk [52]. The same Cochrane Review had mixed results regarding the duration of antibiotic prophylaxis but did see a benefit of a combination of anaerobic and aerobic coverage over either alone or a combination of oral and IV antibiotics over either alone [52].

**Figure 3:**
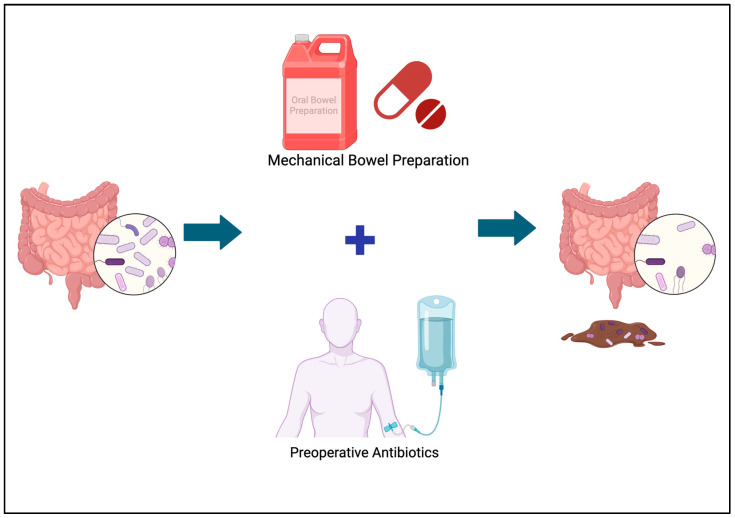
Mechanical bowel preparation and preoperative antibiotics lead to disturbances in the microbiome through an alteration of microbial diversity, changes in ratios of remaining bacteria in the colon, and a loss of bacteria from the mucosa into the stool. Original graphic designed by the authors. Created with BioRender.com (accessed on 20 February 2020).

Although a clear benefit from antibiotics has been established, the benefit of mechanical bowel preparation is less clear. The idea is that if the colon is emptied of stool, there is less risk of contamination intraoperatively and an increased likelihood of a healthy anastomosis. Anastomotic leak is a devastating and potentially fatal complication and is often looked at as the primary outcome to evaluate the benefit of mechanical bowel preparation. The American College of Surgeons National Surgical Quality Improvement Program (ACS NSQIP) data suggests a benefit of mechanical bowel preparation when paired with oral antibiotics compared to no intervention at all [53]. However, two systematic reviews have challenged the effectiveness of mechanical prophylaxis and point to the elevated risks of dehydration, electrolyte imbalance, nausea, vomiting, and renal failure, though there remains a lack of evidence regarding the frequency of these specific outcomes [54,55]. Furthermore, minimal attention has been paid to the unintended consequences of bowel preparation, such as its impact on the gut microbiome and possible further deleterious effects for higher-risk surgical patients such as those with CD. Moreover, research has demonstrated perioperative antibiotics as the most important factor in determining microbial composition postoperatively in some surgical patients [56] [Figure 3].

### The Microbiome and Bowel Preparation

Multiple studies have observed disruption in the gut microbiome related to mechanical bowel preparation [12,57,58,59,60,61,62]. The changes are generally thought to return to normal within two weeks to one month [12,57,58,59]. Gorkiewicz et al. gave patients a three-day course of polyethylene glycol to induce osmotic diarrhea, which resulted in decreased richness of phylotypes [58]. Individuals also had a more similar microbiome to one another after bowel preparation. After induced diarrhea, *Proteobacteria* increased in mucosal samples, while *Faecalibacterium* increased in stool samples [58]. An increase of *Faecalibacterium* in the stool is interpreted as a loss from the mucosa. *Faecalibacterium* is known to produce butyrate and to have anti-inflammatory effects [63]. Additionally, it is less abundant in the colon of patients with CD, and perhaps more interestingly, is decreased in patients with anastomotic leak [63,64]. Although Gorkiewicz et al. studied the microbiome specifically in induced diarrhea, they hypothesize that these changes may be present in any form of osmotic diarrhea, including that which occurs frequently in CD [58]. If *Faecalibacterium* is being lost in the stool because of osmotic diarrhea in an acute flare, perhaps the loss of butyrate production and anti-inflammatory properties perpetuates ongoing inflammation in CD. This begs the question of whether bowel preparation is further increasing the risk of complications in operative CD patients by exacerbating the already existing gut dysbiosis.

The relative abundance of *Firmicutes*, Bacteroidetes, and *Proteobacteria* are frequently implicated in gut dysbiosis [58,60]. *Firmicutes* is present in high concentrations in non-diseased mucosa but decrease significantly with mechanical bowel preparation [58,60,62]. Bacteroidetes is more abundant in stool samples but also present in the mucosa of healthy individuals to a lesser extent than *Firmicutes* [58,62]. After bowel preparation, there is an increase in *Bacteroidetes* such that the relative abundances of it and *Firmicutes* start to approximate each other [58,62]. Also increasing in osmotic diarrhea is *Proteobacteria*, which includes pathogens such as Pseudomonas, Acinetobacter, and Arcobacter [58]. The changes at the phyla level in *Firmicutes* and *Proteobacteria* may still be present at one month. However, at the class level, *Proteobacteria* decreased to below initial levels in one month [60]. Other research has failed to find consistency in the changes in microbiome diversity after bowel preparation; however, it is clear that microbiome changes occur and are likely driven by individual patient factors [61].

Because IBD induces independent changes in the microbiome, Shobar et al. compared the microbiome before and after mechanical bowel preparation in healthy controls and patients with IBD [62]. The IBD sample experienced increased Bacteroidetes after preparation, similar to healthy controls and other studies; however, no significant changes were seen in Actinobacteria or Tenericutes in IBD patients, as seen in healthy controls. While both groups experienced disruption in microbiome composition before and after mechanical bowel preparation, the changes in IBD patients were primarily changes in the fecal samples and seen in both abundant and rare taxa, while changes in healthy patients were primarily in the mucosa and related to rare taxonomic groups. The authors hypothesize that the differences were due to the pre-existing dysbiosis in IBD patients. Interestingly, in both groups, the differences between microbiomes in mucosal and fecal samples became less apparent after bowel preparation, but the IBD patients experienced differences to a greater extent [62]. Induced osmotic diarrhea in IBD patients causes more than just noticeable changes in the microbiome. Colonoscopy preparation is also associated with increased symptoms and even an increase in 5-ASA medications to combat flares in Ulcerative Colitis (UC) patients [62,65]. This was specifically studied in UC, not CD, and cannot be specifically tied to the described microbiome changes, but it is a promising area for future study.

A recent pilot study by Nalluri-Butz et al. compared stool microbial composition in patients after mechanical bowel preparation and colonoscopy with those who underwent surgical bowel preparation and colorectal surgery [12]. The colonoscopy group had significant changes from pre-procedure in the samples gathered during colonoscopy, but these were back to baseline at 10 days [12]. The surgical group had significant changes from preoperative samples in both the intraoperative and 10-day postoperative samples, including increased Enterococcus, Lactobacillus, and Streptococcus. One patient experienced an anastomotic leak and had <1% similarity at 10 days postoperatively from preoperatively [12]. Enterococcus produces collagenase, which has been proposed as a mechanism for anastomotic leak as a result of tissue breakdown [66,67]; Streptococcus and Enterococcus have been associated with dysbiosis as a result of antibiotics [68,69]. The surgical group also experienced a reduction in *Bacteroides*, *Faecalibacterium*, and *Roseburia* [12]. *Faecalibacterium,* being present in lower quantities, has been associated with anastomotic leak [64] and is a known producer of butyrate. In addition to the microbiome changes, butyrate and valeric acid levels were strikingly low after surgery [12].

Surgical bowel preparation has profound impacts on the gut microbiome. Currently, it is applied to most patients undergoing colorectal surgery, including those with CD. The literature is applied to this patient population without specific attention to their needs. The future is promising, however, as the field of medicine moves to a more individualized approach. Someday, patients may have access to a personalized bowel preparation and selective decontamination of pathogens while maintaining or supplementing beneficial organisms [70].

## 7. Perioperative Medication Considerations

Unlike the majority of patients undergoing colorectal surgery for malignancy or diverticular disease, patients with IBD are frequently on medications that suppress the immune system, especially considering a major indication for surgical intervention is a lack of response to medical therapy. Though there are a variety of medical therapies that act by different mechanisms, the common goal is the reduction of inflammation. Other immunosuppressed populations, such as those with diabetes, human immunodeficiency virus, or post-transplantation, are known to have an increased risk of infection and healing complications after surgery. Whether this concept applies to Crohn’s medications has been questioned [71,72,73].

### 7.1. Steroids

Corticosteroids, most often used for acute IBD flares, were the only class of medications to show an increased risk of infectious complications in a Cochrane Review [74]. In particular, there was an increase in intra-abdominal infections, presumably anastomotic leak. Corticosteroids mitigate the stress response, possibly via the microbiome. Bailey 2010 et al. found decreased Bacteroides and increased *Clostridium* in response to catecholamines [47], both of which are implicated in different ways in CD. An increase in *Bacteroides* and a decrease in *Clostridium* would be expected if the downregulation of the stress response impacts the microbiome. A relative increase in *Bacteroides* could be a mechanism by which steroids increase the risk of anastomotic leak [17].

### 7.2. Biologics

Monoclonal antibodies have revolutionized medical treatment for CD, and up to 60% of patients undergoing surgery have received treatment with a tumor necrosis factor (TNF) inhibitor [75]. Initial studies indicated a decrease in surgical intervention for patients on anti-TNF therapy, but rates of surgery over time remain the same [76]. Gaines et al. used a mouse model to evaluate the effects of infliximab on collagenase producing bacteria presence in the gut [77]. Acknowledging that infliximab changes the local bacteriome after colon resection, the authors found no specific increase in collagen producers, which would have mechanistically linked infliximab to anastomotic leak [77]. The previously mentioned 2020 Cochrane Review saw anti-TNF therapy associated with a modest increase in infectious complications postoperatively, but rated the evidence very low certainty [74]. Many authors have attempted to predict responsiveness to anti-TNF therapy, with promising results [78,79]. Although complicated and variable, patterns of microbial composition and metabolomic data are emerging as possible predictors [10,78,79]. Hurych and colleagues compared TNF inhibitors in CD and juvenile idiopathic arthritis (JIA) and found no changes in the fecal microbiome or metabolome for the JIA population, in contrast to the CD patients. In addition, there was no direct effect on in vitro bacterial cultures when exposed to infliximab [10]. These findings indicate that gut microbial changes are not inherent to anti-TNF administration but somehow specific to the inflamed intestine [10]. The microbiome data do not support a mechanism for increased surgical risk surrounding TNF inhibitors and suggest that biologic therapy may actually reduce risk of anastomotic leak by reducing dysbiosis. In the perioperative CD patients, the medical and surgical teams must weigh the risks and benefits for each individual patient.

### 7.3. Pain Management

Patients with CD are susceptible to chronic pain and, therefore, may already be on chronic pain regimens prior to surgery. Opioids were linked to gut dysbiosis in a mouse model by Sharma et al., indicating that chronic opioids may exacerbate inflammation [80]. The mechanisms of dysbiosis were barrier dysfunction, bacterial translocation, tight junction disruption, and increased inflammation [80]. Similarly, Shakhsheer et al. found that morphine treatment increased collagenase-producing *Enterococcus faecalis* near anastomoses [67]. This was associated with increased anastomotic complications [67]. In addition to the general risks of opioid use, dysbiosis is yet another reason to minimize the use of opioids in the perioperative period. One way to do this is to increase preoperative physical activity, optimize nutrition status, and encourage patient engagement with online platforms aiding in opioid cessation, as these can significantly reduce postoperative opioid use and decrease the duration of opioid use [81,82].

In accordance with ERAS protocol, multimodal pain regimens are preferred [30]. This often includes non-steroidal anti-inflammatory agents (NSAIDs). Concerns have also been raised about NSAIDS in the perioperative period and the risk of anastomotic leak [83,84,85,86,87]; however, the data is ambiguous and many studies are flawed [88]. Research regarding the effects of NSAIDs on the gut microbiome is lacking.

### 7.4. Probiotics

Understanding CD in the context of dysbiosis and the varying effects of medications and surgery on the microbiome, probiotics have been suggested as one way to combat dysbiosis. Some studies have promising results [89,90], but the current research is variable in methodology and probiotic regimen. Strains that have shown favorable effects include *Bifidobacterium* spp., *Lactobacillus* spp., *E. coli* Nissle 1917, and *Saccharomyces boulardii* [11]. In particular, VSL-3, which has eight strains of bacteria, has been useful in UC for pouchitis, but so far has not produced results in CD [11,91]. Because of this, ESPEN currently recommends against probiotics for CD [92]. If future microbiota mapping of CD patients is able to establish a more consistent common pattern of dysbiosis, targeted probiotic therapy could have potential as an area of future study. Notably, in contrast to most other interventions, probiotic administration comes with minimal risk and a variety of probiotic supplements are readily available over the counter. Therefore, many patients will inquire about their use and may self-treat with probiotics outside the recommendations of a healthcare provider.

## 8. Discussion and Future Directions

Historically, most of the research on the gut microbiota has been gleaned from stool samples. Some studies are now using mucosal samples [58,62] and/or urine samples [59], but these are limited by small sample sizes. A significant amount of knowledge is also emerging from non-human studies. The changes we have observed in the microbiome need to be further explored by larger studies in the clinical setting. At this point, we are limited to hypothesizing effects based on the presence/absence of certain taxonomic groups, but the true effects of the microbiome makeup are more difficult to elucidate. Metabolomics and proteomics are promising avenues to explore the relationship between dysbiosis and dysfunction. These may lead to a more individualized approach to the perioperative management of patients with CD.

### 8.1. Fecal Microbiota Transplantation (FMT)

Because of its significant role in correcting the gut dysbiosis present in severe *Clostridioides difficile* infection (CDI), FMT is seeing increased interest as a potential treatment for IBD [93]. While it shows promise, the currently available data is limited and variable in methodology [93,94,95]. There are multiple RCTs showing improvements in UC after FMT [95]. However, multiple barriers exist on the pathway to widespread implementation, such as the lack of a standardized administration process, donor selection criteria, preparation, and patient selection. Many of the patients in these studies received FMT for the indication of CDI, not primarily for IBD [93,94]. Despite these barriers, FMT does show promise in UC, but whether this data can be extrapolated to CD patients remains to be seen. There is currently only one published RCT that studies FMT, specifically in CD. The authors note a trend toward remission, but no statistical significance was achieved [96]. FMT is an ongoing area of study, but the current knowledge gaps preclude it from being a recommended treatment for CD.

### 8.2. Surgical Innovations

Efforts to minimize surgical interventions, complete treatments minimally invasively, and prevent complications such as surgical site infections are key in all surgical patients, but especially Crohn’s patients who are predisposed to the negative sequelae of surgery, including dysbiotic shifts in the microbiome. Advancements in endoscopy, as well as localized antibiotic technologies, have shown potential.

Endoscopic balloon dilation, electroincision, and stent placement have emerged as possible treatment options for CD. These techniques can help prevent or relieve obstruction, postpone surgery, and preserve bowel. However, endoscopic intervention in the setting of fibrosis, inflammation, stricturing, fistulae and abscesses is technically challenging, causing complications such as perforation, stent migration, and inadvertent stent incorporation into tissue, making them difficult or impossible to remove [97]. Stents are not a feasible long-term solution in CD; however, newer stents are softer and more flexible, causing less inflammation and fibrosis. Scotti et al. conducted a systematic review of endoscopic stenting in stricturing CD and found that partially covered self-expanding metal stents can be considered to help delay surgery for short stricture segments [98]. However, stents must have planned retrieval and are not suitable for long-term treatment [98]. Overall, data is limited, and most studies are retrospective in nature.

Technologies targeted at localized control of microbial burden could help shift use away from the systemic antibiotic therapy in the perioperative setting that creates profound dysbiosis. Two technologies that could be evaluated for efficacy in this domain include triclosan-coated (TCS) sutures and antibiotic irrigating wound protectors, both initially developed to reduce the risk of SSIs. Evidence for TCS use is mixed, but two meta-analyses suggest clinical effectiveness in decreasing SSI [99,100]. In a colorectal-specific simulated animal model, Suh et al. showed the efficacy of a novel surgical device that used intraoperative antibiotic irrigation to prevent SSI [101]. Their in vivo study showed the safety and efficacy of the device in preventing intraoperative wound contamination [101], and further meta-analysis investigating such technologies found that SSIs were significantly lower with wound protectors in lower gastrointestinal surgery compared to no wound protectors [102]. Further research needs to be completed to assess if controlling microbial burden at the local level in surgical patients could allow safe and efficacious reduction of systemic perioperative antibiotics.

## 9. Conclusions

It is clear that there is a relationship between the gut microbiome and CD and that many factors, including surgery, diet, and medications, disrupt the microbiota. Dysbiosis is common in Crohn’s patients and may be exacerbated by malnutrition, steroids, narcotic use, and diarrhea. Dysbiosis is also a major risk factor for anastomotic leak, and special attention should be paid to Crohn’s patients in this regard. Antibiotic use is widespread in the United States, and CD patients may be more commonly prescribed antibiotics in comparison to the general population due to the known infectious complications of the disease, and they also have added exposure from perioperative use. As a known risk factor for dysbiosis, it is imperative to exercise judicious use of antibiotics in this population. Continued advances in surgical technology are opening new avenues to minimize dysbiosis.
